# Minimum imaging dose for deep learning-based pelvic synthetic computed tomography generation from cone beam images

**DOI:** 10.1016/j.phro.2024.100569

**Published:** 2024-03-22

**Authors:** Yan Chi Ivy Chan, Minglun Li, Adrian Thummerer, Katia Parodi, Claus Belka, Christopher Kurz, Guillaume Landry

**Affiliations:** a Department of Radiation Oncology, LMU University Hospital, LMU Munich, Munich 81377, Germany; b Department of Radiation Oncology, Lueneburg Hospital, Lueneburg 21339, Germany; c Department of Medical Physics, Faculty of Physics, Ludwig-Maximilians-Universität München, Garching b. München 85748, Germany; d German Cancer Consortium (DKTK), partner site Munich, a partnership between DKFZ and LMU University Hospital Munich, Munich 81377, Germany; e Bavarian Cancer Research Center (BZKF), Munich 81377, Germany

**Keywords:** Deep learning, Generative networks, Synthetic CT, Low dose CBCT, Online adaptation, cycleGAN, Contrastive unpaired translation

## Abstract

**Background and purpose:**

Daily cone-beam computed tomography (CBCT) in image-guided radiotherapy administers radiation exposure and subjects patients to secondary cancer risk. Reducing imaging dose remains challenging as image quality deteriorates. We investigated three imaging dose levels by reducing projections and correcting images using two deep learning algorithms, aiming at identifying the lowest achievable imaging dose.

**Materials and methods:**

CBCTs were reconstructed with 100%, 25%, 15% and 10% projections. Models were trained (30), validated (3) and tested (8) with prostate cancer patient datasets. We optimized and compared the performance of 1) a cycle generative adversarial network (cycleGAN) with residual connection and 2) a contrastive unpaired translation network (CUT) to generate synthetic computed tomography (sCT) from reduced imaging dose CBCTs. Volumetric modulated arc therapy plans were optimized on a reference intensity-corrected full dose CBCT_cor_ and recalculated on sCTs. Hounsfield unit (HU) and positioning accuracy were evaluated. Bladder and rectum were manually delineated to determine anatomical fidelity.

**Results:**

All sCTs achieved average mean absolute mean absolute error/structural similarity index measure/peak signal-to-noise ratio of ⩽59HU/⩾0.94/⩾33 dB. All dose-volume histogram parameter differences were within 2 Gy or 2%. Positioning differences were ⩽0.30 mm or 0.30°. cycleGAN with Dice similarity coefficients (DSC) for bladder/rectum of ⩾0.85/⩾0.81 performed better than CUT (⩾0.83/⩾0.76). A significantly lower DSC accuracy was observed for 15% and 10% sCTs. cycleGAN performed better than CUT for contouring, however both yielded comparable outcomes in other evaluations.

**Conclusion:**

sCTs based on different CBCT doses using cycleGAN and CUT were investigated. Based on segmentation accuracy, 25% is the minimum imaging dose.

## Highlights

• Investigating the minimum CBCT imaging dose for pelvic synthetic CT generation.
• Comparing two AI algorithms to generate sCT from dose reduced CBCT (25%, 15%, 10%).
• Evaluating sCT in terms of metrics relevant to CBCT-guided adaptive radiotherapy.
• 25% dose as minimum for accurate VMAT dose calculation and organ delineation.

## Graphical abstract

**Figure:**
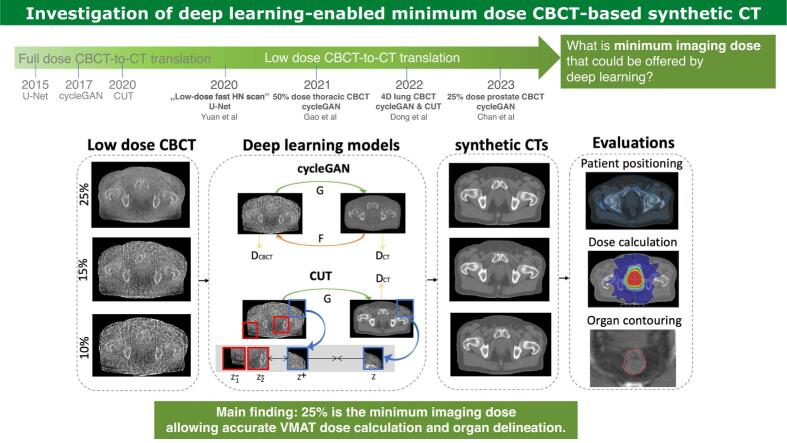

## 1 Introduction

In image-guided radiotherapy (IGRT), deep learning (DL) algorithms have been widely employed to enhance radiotherapy treatments. Particularly for the pelvic region, where the anatomy exhibits inter- and intra-fractional variations, the adaptive workflow relies on accurate cone beam computed tomography (CBCT)-to-CT translation [1], [2], and organ segmentation on synthetic CTs (sCT) [3], [4]. CBCT imaging dose has often been disregarded, viewed as negligible compared to the therapeutic dose. However, studies suggested that daily CBCT potentially results in considerable additional organ doses in the pelvic region [5], [6], [7]. Each pelvic scan can deliver up to 22.7 mSv effective dose [8].

Adhering to “as low as reasonably achievable” (ALARA), radiation oncologists use the lowest possible imaging dose or restrict the frequency of CBCT scans to reduce secondary cancer risk. Further reducing imaging dose, however, remains impractical since the image quality would degrade to unusable levels with potential loss of anatomical information. Lower imaging dose CBCTs with enhanced image quality could not only mitigate the secondary cancer risk concern, but also offer a higher flexibility in terms of in-room imaging frequency and enable online treatment dose adaptation. With sufficient sCT quality, one could also avoid acquiring new planning CTs for plan adaptation, thus further reducing imaging dose.

DL-enabled CBCT-to-CT translation has mostly been developed for standard full dose CBCT. Three DL architectures have been applied to pelvic scans: U-Net [9], cycleGAN [10] and contrastive unpaired translation (CUT) [11]. U-Nets were trained with paired data in image [12], [13], [14] or projection domain [14], [15], [16], [17]. To overcome potential misalignments, cycleGAN has been used for unpaired training [18], [19], [20], [21], [22], [23]. In recent studies [24], [25], [26], CUT demonstrated better performance over cycleGAN. Treatment dose calculation on CUT, however, remained unexplored.

Limited studies explored the possibility of using low imaging dose CBCT. Our previous study [27] investigated the feasibility of removing under-sampling artifacts and correcting intensities of 25% imaging dose CBCT using cycleGAN. sCT from 25% imaging dose CBCT (≈0.6 mGy) showed high accuracy in therapeutic photon dose calculation, anatomical fidelity (in terms of Dice similarity coefficient (DSC) and Hausdorff distance (HD) of contours) and positioning. In [24], cycleGAN and CUT removed streaks from 4D CBCT which is comparable to low dose CBCT. There are a few low dose CBCT-to-CT studies in other anatomies [28], [29]. Among low dose CBCT-to-CT studies, there is a scarcity of systematic investigation of the maximum imaging dose reduction level that DL could offer. In most CBCT-to-CT studies, organ segmentation is rarely evaluated except [18], [21], [22] in pelvic and [30] head and neck region.

In this study, we aim at finding the achievable lowest imaging dose using cycleGAN and CUT in terms of all metrics relevant to CBCT-guided adaptive radiotherapy: image quality, positioning, organs-at-risk (OAR) contouring accuracy and therapeutic photon dose calculation. We investigated imaging dose levels in terms of sCT generation from a CBCT with reduced number of projections (25%, 15% and 10%) by removing under-sampling artefacts and correcting image intensities. Dose reduction is achieved via the reduction of the number of projections.

## 2 Materials and methods

**Fig. 1:**
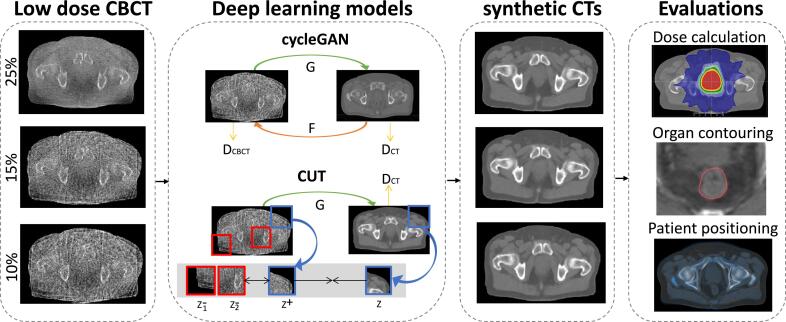
Workflow of CBCT restoration at different dose reduction levels investigated in this study. Low dose CBCTs were set as inputs in the cycleGAN and CUT algorithms to generate sCTs, which were then evaluated by means of patient positioning, dose calculation and contouring accuracy. The black arrows denote the sequence of the investigation steps in this study (low dose CBCT generation, deep learning model training, sCT generation, and finally the evaluation of therapeutic photon dose calculation, contouring and positioning). Models were trained separately for each dose reduction level. G and F denote generators, D_CBCT_ and D_CT_ denote discriminators, z denotes the image patches used in CUT.

The workflow of CBCT restoration at different dose levels is illustrated in Fig. 1. In general, imaging dose was reduced by retroactively reducing the number of acquired projections.

### 2.1 Patient database for model training

The database contained 41 prostate cancer patients who received volumetric modulated arc therapy (VMAT) at Department of Radiation Oncology of LMU University Hospital. One planning CT (pCT) acquired before treatment course and CBCT images of one arbitrary fraction of each patient were collected.

Bavarian state law (Bayrisches Krankenhausgesetz/Bavarian Hospital Law §27 Absatz 4 Datenschutz) allows the use of patient data for research, provided that any person’s related data are kept anonymous. All patient data were fully anonymised. Identification from pelvic CT data is not possible. German radiation protection laws request a regular analysis of outcomes in the sense of quality control and assurance, thus in the case of purely retrospective studies no additional ethical approval is needed under German law.

Only CBCT datasets acquired with the lowest dose pelvic protocol (120 kV tube voltage, 20 ms exposure time, 20 mA X-ray tube current per projection) in treatment position using the XVI system (version 5.52) of a Synergy medical linear accelerator (Elekta, Sweden) were selected. For each fully sampled (FS) scan, approximately 350 projections [346, 357] were acquired over 360° with a shifted panel and reconstructed using Feldkamp–Davis–Kress (FDK) implementation of Reconstruction ToolKit (RTK) [31], referred to as CBCT_FS_. To investigate achievable dose reduction levels, CBCTs were under-sampled to 25% (∼90 projections), 15% (∼52 projections) and 10% (∼35 projections) and reconstructed using the same settings. Since each projection was acquired with a fixed dose, reducing the number of projections results in a dose reduction.

pCTs were acquired without contrast agent on a Toshiba Acquilion LB CT scanner (Canon Medical Systems, Japan). A virtual CT (vCT) was generated using a dedicated deformable image registration (DIR) algorithm mapping the pCT onto the daily CBCT_FS_ [32]. For reference, a intensity-corrected CBCT_cor_ was generated using a projection-based scatter correction technique [14], [17], [33], [34], [35], [36] based on DIR of pCT to CBCT and forward projection followed by conjugate gradient iterative reconstruction [31].

CBCTs, vCTs and CBCT_cor_ were padded to an axial size of 512 × 512 pixels (1 mm × 1 mm) with a slice thickness of 1 mm. Details of the data acquisitions and pre-processing can be found in the Supplementary material.

### 2.2 Deep learning algorithms

Low dose CBCT-to-CT translation can be formulated as:

$$\mathrm{sCT}=G\left({\mathrm{CBCT}}_{\mathrm{LD}}\right)$$

where G is an encoder-decoder based model that simultaneously converts CBCT_LD_ to sCT while preserving the anatomical content. In this study, cycleGAN and CUT algorithms were employed to train G.

#### 2.2.1 cycleGAN

We applied the cycleGAN algorithm that was implemented in a previous study [27]. This training process involved two sets of generator and discriminator networks. A cycle consistency loss ($L_{\mathrm{cyc}}$) is computed to stabilise anatomical mappings between CBCT and CT. A residual skip connection was used for both generators to attain higher anatomical fidelity. vCT was used in the training instead of pCT to evaluate the efficacy of an additional paired loss term.

#### 2.2.2 CUT

We adapted the CUT algorithm proposed by Park et al.[11]. Only one set of generator and discriminator is required, since $L_{\mathrm{cyc}}$ is replaced by a patchwise contrastive loss ($L_{\mathrm{PatchNCEx}},L_{\mathrm{PatchNCEy}}$). As shown in Fig. 1, a sCT patch should match more with its corresponding input CBCT patch (denoted as positives), in comparison with other random CBCT patches (denoted as negatives). The encoder part of the generator (G_enc_) followed by a two-layer multilayer perceptron (MLP) network is employed, which allows the model to learn and project both patches to a shared feature embedding space.

Training of each model used identical data, pre-processing and data augmentation. Details are provided in Supplementary material.

### 2.3 Training details

For each of the CBCT dose reduction levels, cycleGAN and CUT models were trained with 4-fold cross-validation with 25 out of 30 patients per fold, from which the median of the four predicted images was used. We determined hyper-parameters for each model through ensemble validation on three patient datasets. Subsequently, we preserved the model weights associated with the highest validation performance and applied them for testing.

The test set consisted of 8 patient datasets. The generators for every imaging dose level were applied to convert CBCTs into sCTs. Details are provided in Supplementary material.

### 2.4 Evaluation

#### 2.4.1 Image quality

sCTs of different imaging dose levels for the test set were compared to CBCT_cor_ in terms of the mean absolute error (MAE), mean error (ME), structural similarity index measure (SSIM) and peak signal-to-noise ratio (PSNR). Only voxels within the joint body outline of CBCT_cor_ and sCTs were included.

#### 2.4.2 Treatment dose calculation

VMAT plans on CBCT_cor_ for the test patients were generated in a research version of a commercial treatment planning system (TPS) (RayStation, version 10.01, RaySearch, Sweden). Contours of target structures and OAR were transferred from the pCT to sCTs and CBCT_cor_ using DIR, VMAT plans were optimized on an isotropic dose grid of 3.0 mm using a collapsed-cone dose engine. These plans were then recalculated on all sCTs. The prescription was 74 Gy in 37 fractions and we aimed at a clinical target volume (CTV) $V_{95\%}$ of 100%, and planning target volume (PTV) $V_{95\%}$ better than 95% of the prescription dose. The dose-volume histogram (DVH) constraints for the bladder and the rectum were pursued as suggested in the QUANTEC report [37]. The VMAT dose distributions were compared with the CBCT_cor_ reference considering DVH parameters of clinically relevant target structures and OAR. CTV and PTV $D_{98\%}$ and $D_{2\%}$, together with PTV $D_{50\%}$ and $V_{95\%}$ were analyzed. For the bladder $V_{60/65\;\mathrm{Gy}}$ and for the rectum $V_{50/60/65\;\mathrm{Gy}}$ were analyzed. Moreover, the voxels passing a therapeutic dose difference (DD) analysis with a 1% and 2% criterion (10% threshold) were compared. For each dose parameter, results from sCTs were compared to CBCT_cor_ using Wilcoxon signed-rank tests. Similarly, low imaging dose sCTs were compared to FS sCT for both models. P-values less than 0.05 were considered significant.

#### 2.4.3 Segmentation accuracy

To determine the anatomical fidelity of all sCTs, bladder and rectum were contoured manually under the supervision of a radiation oncologist using the research TPS on CBCT_FS_ and sCTs. Please keep in mind that these contours were unrelated to the contours used to generate the treatment plans used for the treatment dose evaluation of Section 2.4.2. DSC, average and $95^{\mathrm{th}}$ percentile HD (HD_avg_, HD_95_) of the contours of all sCTs were compared with CBCT_FS_ as reference. sCTs from all imaging dose reduction levels, as well as from both models at the same dose reduction, were statistically analysed using Wilcoxon signed-rank tests.

#### 2.4.4 Positioning accuracy

To evaluate positioning accuracy at different CBCT dose reduction levels, all sCT images were rigidly registered to the pCT using TPS (automated, gray level, six degrees of freedom). The transformations were compared to the one obtained from registering CBCT_FS_ to pCT.

## 3 Results

The average time to generate a sCT slice from CBCT was 6 ms for both models. Detailed epoch selection and the corresponding training time are shown in Supplementary Table 1.

### 3.1 Image comparison

**Fig. 2:**
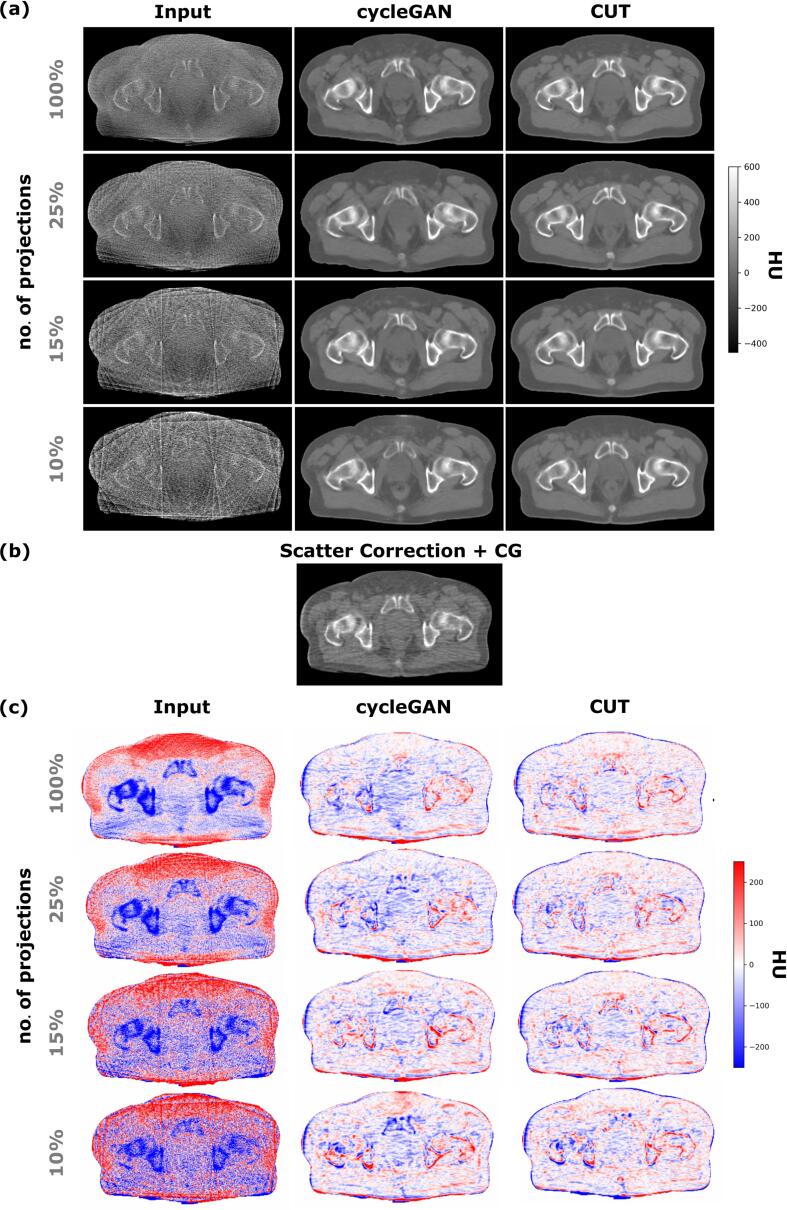
Axial view of (a) the CBCT inputs and sCTs generated by cycleGAN and CUT with 100%, 25%, 15%, 10% projections; (b) the scatter corrected CBCT_cor_ reconstructed with conjugate gradient (CG); (c) HU difference between inputs and corresponding sCTs with CBCT_cor_ of a test patient.

Fig. 2 illustrates sCTs, CBCT_cor_ of a representative test patient and their corresponding HU differences. Both cycleGAN and CUT removed streak artifacts from all CBCTs, and simultaneously converted them into diagnostic quality. Compared to inputs, all sCTs show reduced differences to CBCT_cor_. The remaining differences are observed at body outline and bone interfaces. The coronal view is illustrated in Supplementary material Fig. 1.

All metrics are substantially enhanced by both models (Supplementary Table 2). The average MAE of all sCTs with respect to CBCT_cor_ were improved from ⩾102 HU to ⩽59 HU. The average ME of the majority of the sCTs has decreased by ⩾7 HU. SSIM/PSNR were enhanced from ⩽0.91/⩽33 dB on CBCTs to ⩾0.94/⩾33 dB on sCTs.

### 3.2 Treatment dose calculation

**Fig. 3:**
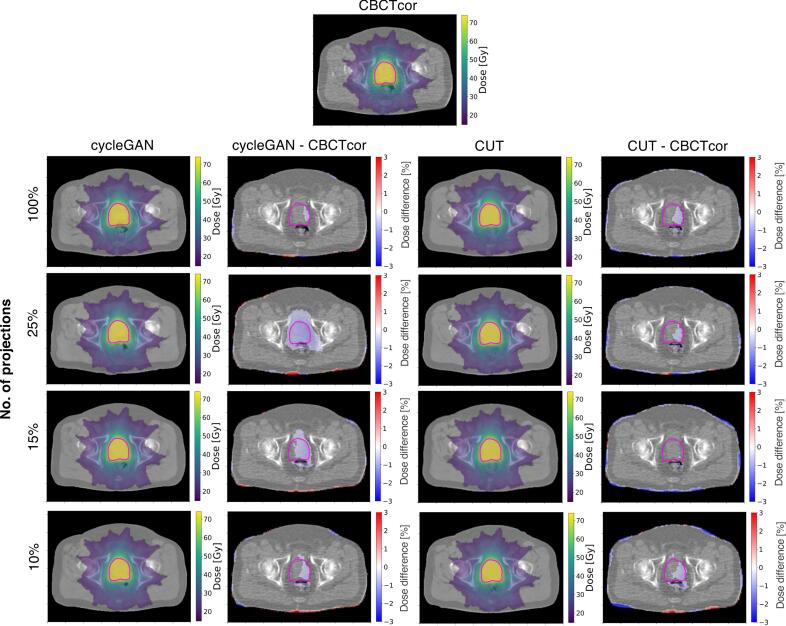
VMAT dose distributions of an exemplary patient. Dose distributions optimized on CBCT_cor_ and recalculated on sCTs at difference dose reduction levels, and their corresponding differences for cycleGAN and CUT. The PTV is shown in magenta. Dose differences below 0.4% are not shown for better visualization.

Fig. 3 shows the treatment dose distribution and difference of an exemplary test patient. Compared sCTs to CBCT_cor_, only minor dose differences were found in the PTV region (<3%). The remaining treatment dose differences were mainly in patient outline.

**Fig. 4:**
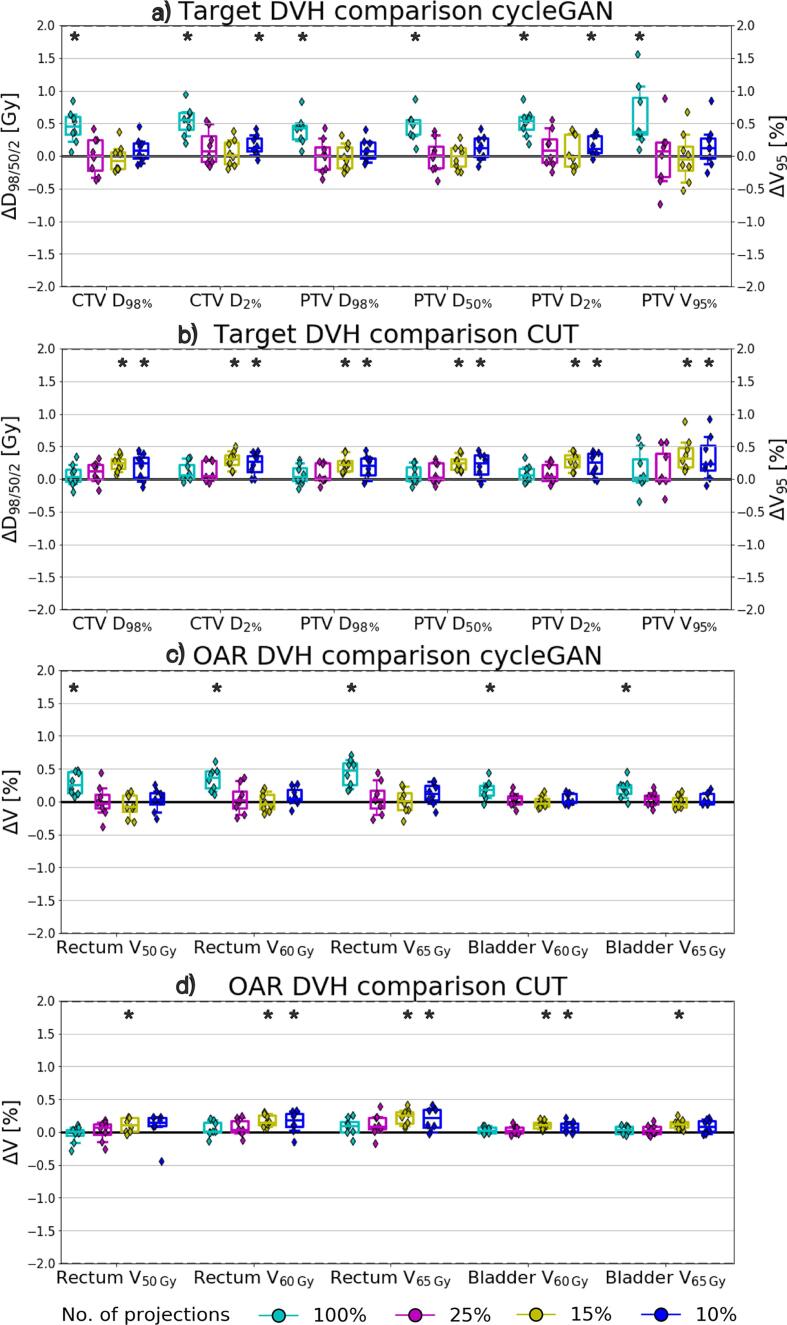
Clinically relevant DVH parameter differences of sCTs in different dose reduction levels with respect to CBCT_cor_ for target and OAR structures using (a, c) cycleGAN and (b, d) CUT. Each data point represents a test patient. Whiskers correspond to the 5th–95th percentile. The box denotes the interquartile range, and a horizontal line inside the box is used to represent the median. All values correspond to the total treatment dose of the fractionated treatment. Significant difference between sCT and CBCT_cor_ is indicated by a star (p-value <0.05).

In Fig. 4, target and OAR DVH parameter differences with respect to CBCT_cor_ over all test patients are depicted. Deviations were within 2 Gy for dose DVH parameters ($D_{2/50/98\%}$) and below 2% for volume DVH parameters ($V_{50/60/65\;\mathrm{Gy}}$). Particularly in the target DVH comparison, the mean differences of $D_{2/50/98\%}$ comparing all sCTs with respect to CBCT_cor_ were ⩽0.5% for the PTV. For cycleGAN, no significant differences were found in the majority of the low dose sCT, except CTV $D_{2\%}$ and PTV $D_{2\%}$ of the 10% sCT. Statistically significant differences were observed for all FS sCTs, but most magnitudes were constrained by 1 Gy. For CUT, significant differences were observed in 15% and 10% sCTs for most of target and OAR DVH parameters, except rectum $V_{50\%}$ and bladder $V_{65\%}$ for 10% sCTs.

**Fig. 5:**
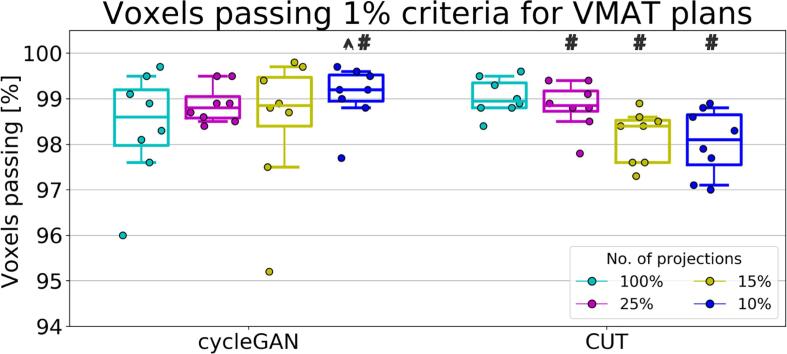
Voxels passing a 1% dose difference criterion in the eight test patients for the VMAT plans recalculated on sCTs from different dose reduction levels using cycleGAN and CUT with respect to CBCT_cor_. Each data point represents a test patient. Whiskers correspond to the 5th–95th percentile. The box denotes the interquartile range, and a horizontal line inside the box is used to represent the median. Significant difference comparing dose reduced sCT to FS sCT is indicated by a hash sign (p-value <0.05). Significant difference comparing cycleGAN and CUT for identical dose sCT is indicated by a circumflex (p-value <0.05).

Fig. 5 illustrates the quantitative results of the treatment dose difference analysis of the VMAT plans comparing sCTs to CBCT_cor_ with a 1% criterion. The average 1% DD pass-rates of all sCTs were above 95% for cycleGAN and 97% for CUT. Statistically significant differences were observed comparing the dose reduced sCTs to the FS sCT for CUT, the 10% sCT to the FS sCT for cycleGAN. CycleGAN performed significantly better than CUT for 10% sCT. The average 2% DD pass-rates were higher than 98% for both models, indicating an excellent agreement of all sCTs to the reference CBCT_cor_.

### 3.3 Anatomical accuracy

**Fig. 6:**
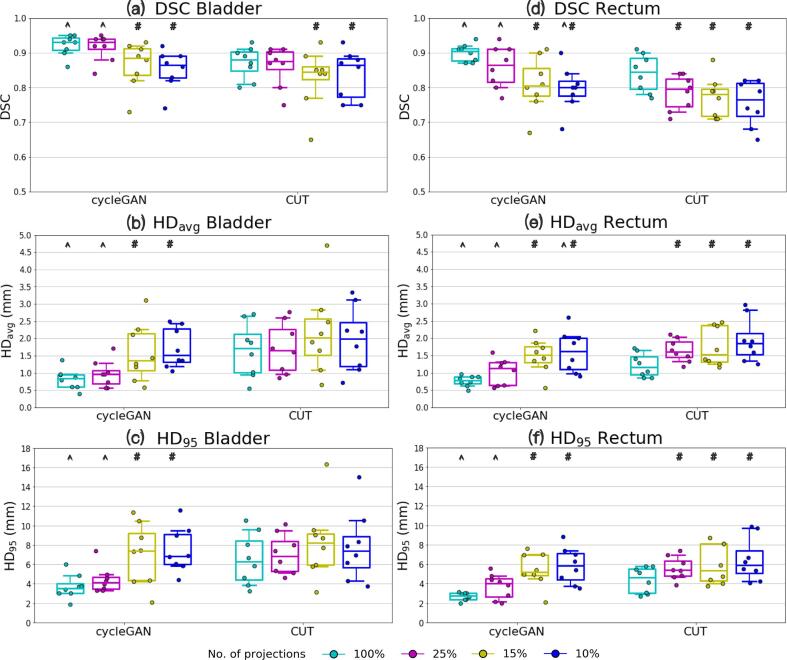
The anatomical fidelity results of (a–c) bladder and (d–f) rectum in terms of Dice similarity coefficient (DSC), average and 95th percentile Hausdorff distance (HD_avg_, HD_95_) comparing sCTs with CBCT_FS_ in the test patients. Each data point represents a test patient. Whiskers correspond to the 5th–95th percentile. The box denotes the interquartile range, and a horizontal line inside the box is used to represent the median. Significant difference comparing dose reduced sCT to FS sCT is indicated by a hash sign (p-value <0.05). Significant difference comparing cycleGAN and CUT for identical dose sCT is indicated by a circumflex (p-value <0.05).

Fig. 6 shows the (a-c) bladder and (d-f) rectum contouring results. For bladder, the average DSC was above 0.80 in all sCTs with respect to CBCT_FS_. HD_avg_/ HD_95_ of bladder were ⩽1.5 mm/⩽8.0 mm for cycleGAN, and ⩽2.0 mm/⩽8.3 mm for CUT in all sCTs. For cycleGAN, significant differences were observed comparing 15% and 10% to FS sCTs in all metrics. For CUT, significant differences only in DSC were observed comparing 15% and 10% to FS sCTs. CycleGAN performed significantly better than CUT in FS and 25% sCT for all metrics.

For rectum, the average DSC was ⩾0.80/⩾0.75 for cycleGAN/CUT in all sCTs. HD_avg_/ HD_95_ of rectum were ⩽1.7 mm/⩽6.0 mm for cycleGAN, and ⩽1.9 mm/⩽6.6 mm for CUT. For cycleGAN, significant differences were observed comparing 15% and 10% to FS sCTs in all metrics. For CUT, significant differences were observed comparing 25%, 15% and 10% to FS sCTs. CycleGAN performed significantly better than CUT in FS, 25% and 10% sCT for all metrics except HD_95_.

In both organs, FS sCT has the highest DSC, lowest HD_avg_ and HD_95_ among all sCTs for cycleGAN and CUT. In addition, bladder had higher DSC and lower HD_avg_ and HD_95_ than rectum in both models.

### 3.4 Positioning accuracy

Compared to CBCT_FS_-to-pCT rigid registration, the mean absolute differences of rigid transformation parameters in all sCTs-to-pCT registrations were less than 0.30 mm or 0.30° for both models, demonstrating sCTs from the investigated low dose CBCT have the potential to align patients accurately. Detailed results are provided in Supplementary material Table 3.

## 4 Discussion

We investigated sCT generation based on different CBCT imaging dose reduction levels achieved by reducing the number of projections using cycleGAN and CUT, and evaluated image quality, dose calculation and organ segmentation accuracy. The CBCT inputs were initially reconstructed with 100%, 25%, 15% and 10% projections. Our primary objective was to determine the largest imaging dose reduction without loss of accuracy.

Over the evaluation metrics for image quality, treatment dose calculation and positioning accuracy, limited deviations were noted among all imaging dose reduction levels. However, organ segmentation showed differentiation among the sCTs for both models. From the DSC, HD_avg_ and HD_95_ results (Fig. 6), the performance drops at 15% dose, where the cycleGAN model began to exhibit degraded accuracy in generating accurate bladder and rectum shapes, as observed from the significant differences appearing at 15% and 10% dose sCT compared to CBCT_FS_. For CUT, the performance declined at 15% dose for the bladder, and already at 25% for the rectum. In addition, as revealed by the significant differences in all metrics for organ contours, cycleGAN performed slightly better than CUT in FS and 25% dose, while in further reduced dose levels both models demonstrated similar inferior performance.

Compared to previous pelvic sCT segmentation studies [18], [21], [22], this is the first time sCTs based on CBCTs at different imaging dose levels were compared. A direct comparison to other studies is not easily possible because the imaging dose of CBCT testing data is different. However, mean DSC of bladder and rectum for FS sCT, from cycleGAN (0.92, 0.90) and CUT (0.88, 0.84) agree with the other studies (0.89–0.92, 0.81–0.87) [18], [21], [22]. No rectum DSC was reported in [18].

Regarding therapeutic dose accuracy, DVH parameters difference were within 2 Gy or 2%, which aligned with previous studies using cycleGAN [20], [27]. Photon dose calculation using CUT is however not yet found in other studies. For CUT, the drop of performance at 15% sCTs was manifested for OAR DVH parameters. Using 1% DD criteria, we observed a significant decrease of voxels passing at 10% for cycleGAN and 25% for CUT. However all values were still above 97%, indicating high agreement which allowed accurate dose calculation.

Regarding image quality, both models substantially enhanced all CBCTs and CUT performed slightly better than cycleGAN. Coronal views showed slight jittering in the internal organs along slices, since the training was conducted in 2D. Compared to [24], our sCTs yielded higher PSNR and SSIM but higher MAE. These differences are mainly due to the use of a deformed CT as reference in [24], which might have more uncertainties from DIR but less scatter noise than our reference CBCT_cor_.

While this study illustrated minimum imaging dose at 25%, it is limited by the number of patients in the test datasets. Besides, the models are not anatomical-site-agnostic as only pelvic datasets were used. Moreover, DL-generated images may suffer from anatomical inaccuracies. Despite the use of $L_{\mathrm{PatchNCEx}}$ and $L_{\mathrm{PatchNCEy}}$ in CUT, accurately predicting organs, particularly in those with variable shapes like rectum, remains challenging. Low dose CBCTs can yield high positioning accuracy [38] or a small dosimetric deviation using a water-density override. However, it is still meaningful to generate sCTs which enable organ contouring for adaptation.

Unlike prior DL-enabled CBCT-to-CT works [12], [13], [14], [15], [16], [18], [19], [20], [21], [23], [24], [25], [26], this study investigated anatomical fidelity in sCT by manual OAR contouring. This aspect revealed a performance threshold for imaging dose reduction. Our results suggest that a CBCT imaging dose as low as 25% is clinically feasible, enabled by the optimized cycleGAN or CUT model. Further reduction to 15% or 10% requires additional DL advancements.

sCTs based on different CBCT imaging dose reduction levels (100%, 25%, 15% and 10%) using cycleGAN and CUT were investigated. While all sCTs demonstrated very good dosimetric, HU and positioning accuracy for both models, considerable differences were found in terms of contouring accuracy. In line with all evaluations, 25% is the minimum imaging dose without loss of anatomical accuracy.

## CRediT authorship contribution statement

**Yan Chi Ivy Chan:** Conceptualization, Data curation, Methodology, Investigation, Software, Formal analysis, Writing – original draft, Visualization. **Minglun Li:** Data curation, Writing – review & editing. **Adrian Thummerer:** Methodology, Software, Writing – review & editing. **Katia Parodi:** Writing – review & editing. **Claus Belka:** Writing – review & editing. **Christopher Kurz:** Conceptualization, Data curation, Methodology, Software, Writing – review & editing, Supervision. **Guillaume Landry:** Conceptualization, Data curation, Methodology, Software, Writing – review & editing, Supervision.

## Declaration of Competing Interest

The authors declare the following financial interests/personal relationships which may be considered as potential competing interests: The Department of Radiation Oncology of LMU University Hospital of LMU Munich has a research agreement with Elekta.
