# Supplementary material for: Minimum imaging dose for deep learning-based pelvic synthetic computed tomography generation from cone beam images
Source: Phys Imaging Radiat Oncol. 2024 Mar 22;30:100569. doi: 10.1016/j.phro.2024.100569 (PMC11519690; doi:10.1016/j.phro.2024.100569)
Supplement: Supplementary data 1 [file mmc1.pdf]

Supplementary material

Data acquisition and pre-processings

planning CT (pCT)s of the selected prostate cancer patients were reconstructed with an image grid of 1.074 mm × 1.074 mm × 3.000 mm in combination with a 55 cm lateral field of view (FOV). In each fraction, these patients were advised to follow an in-house bladder and rectum filling protocol before treatment and cone beam computed tomography (CBCT) scanning. Their corresponding CBCT images were acquired with an increased lateral FOV by using a laterally-shifted detector panel in M position and a bow-tie filter. Images with body outline truncation in spite of the increased FOV were excluded from the study. The mean, min, max time gap between pCT and CBCT are 26, 7, 61 days respectively. CBCT inputs ($\mathrm{CBCT_{FS}}$, $\mathrm{CBCT_{LD25}}$, $\mathrm{CBCT_{LD15}}$ and $\mathrm{CBCT_{LD10}}$) were reconstructed identically using Feldkamp–Davis–Kress (FDK) (as described previously in [1]) with 410 × 410 × 264 voxels on an isotropic $1.0\,\mathrm{mm}^3$ grid. The patient couch was removed from the CBCT input images by thresholding and morphological maskings, followed by zero padding to an image size of 512 × 512 pixels. The pixel intensity of all CBCTs was normalised in the attenuation coefficient value (μ) range [0, 0.04] (values above 0.04 were set to 0.04).

vCTs were generated by registering the pCT to the daily CBCT via DIR. As described in [2], we aim for 1) image similarity which is computed by normalized gradient fields, and 2) deformation regularity which is computed by curvature regularization. The optimization problem is solved in a discretize-then-optimize scheme using a quasi-Newton L-BFGS optimizer. Following the generation, vCTs were re-sampled to an isotropic $1.0\,\mathrm{mm}^3$ grid and an image size of 512 × 512 pixels. The table was also removed. The pixel intensity was empirically converted to the range of the CBCT images ((Hounsfield units (HU) + 1024) / 65536) [3]. The resulting intensities were clipped to the range [0, 0.05] (values above 0.05 were set to 0.05).

The reference $\mathrm{CBCT_{cor}}$ were generated (as described in [4]) and reconstructed using iterative conjugate gradient (CG) with 410 × 410 × 264 voxels on an isotropic $1.0\,\mathrm{mm}^3$ grid. In the CG algorithm [5], the objective function consists of a data consistency term, Laplacian and Tikhonov regularization as shown in the following formulation:

$$||\mathrm{sqrt}(D)(Rf-p)||_2^2 + \gamma||\nabla \cdot f||_2^2 + \mathrm{T}||f||_2^2 \qquad \text{(S1)}$$

with R the forward projection operator, f the image to be reconstructed, p the measured projections, D the displaced detector weighting operator, γ the weighting of the Laplacian regularization, ∇ being the spatial derivative of the image f, T being the strength of the Tikhonov regularization. The goal of this iterative CG algorithm is to find the image f that minimizes the above equation. By empirical experiments, the hyper-parameters for reconstructing the CBCT images were set to 100 iterations, $\gamma = 1000$ and T = 100 for the optimal image quality.

The beginning and last 35 image slices of all CBCT inputs, $\mathrm{CBCT_{cor}}$ and vCT in superior–inferior direction were excluded due to partial FOV cone truncation.

Deep learning algorithms

Two deep learning algorithms were used to investigate each of the dose reduction levels in CBCT-to-CT translation tasks. Both algorithms are based on generative adversarial networks (GAN) and trained with a back-and-forth interaction between a generator and a discriminator. An adversarial loss term $L_{\mathrm{adv}}(\mathrm{G}, \mathrm{D}, \mathrm{CBCT}, \mathrm{CT})$ is computed in both algorithms for which the generator G tries to convert low dose CBCT to synthetic CT (sCT) such that is indistinguishable from real CT according to the discriminator D.

- cycleGAN: the first algorithm was the cycleGAN that we implemented in a previous study [1]. In addition to the 25% dose CBCT, 15% and 10% dose CBCTs were specified as the inputs to train corresponding sets of generators and discriminators in this study. To attain higher anatomical fidelity, a residual skip connection was added for both generators to keep the high resolution features in the input image and reduce the vanishing gradients problem in the encoding process. This approach has been reported to improve geometric fidelity in the field of histopathology [6] and was used in a previous CBCT-to-CT study [1]. A cycle consistency loss L_{cyc} is introduced to stabilise the anatomical mappings between CBCT and CT using L1 norm regularisation. This process involves training an extra set of generator and discriminator, for which CT and CBCT are swapped. vCT was used in the training, as we added an L2 norm between CBCT and corresponding vCT to investigate the efficacy of such a paired loss. The total objective function

can be formulated as

$$\begin{aligned} L_{\text{cycleGAN}}(\mathrm{G}, \mathrm{F}, \mathrm{D}_{\text{CBCT}}, \mathrm{D}_{\text{CT}}) = & L_{\text{adv}}(\mathrm{G}, \mathrm{D}_{\text{CT}}, \text{CBCT}, \text{CT}) + \\ & L_{\text{adv}}(\mathrm{F}, \mathrm{D}_{\text{CBCT}}, \text{CT}, \text{CBCT}) + \\ & \lambda_1 L_{\text{cyc}}(\mathrm{G}, \mathrm{F}) + \\ & \lambda_2 L_{\text{L2}}(\text{CBCT}, \text{CT}). \end{aligned} \tag{S2}$$

where G and F denote the generators in forward and backward cycle respectively, D_{CBCT} and D_{CT} denote the discriminators, λ_1 and λ_2 are hyperparameters that were empirically set to 25 and 0 for the optimal image quality in this study.

- CUT: the second algorithm is an alternative one-side translation to reduce the computing resources of the auxiliary network. This can be achieved by replacing L_{cyc} with a loss on image patches, referred to as patchwise contrastive loss L_{PatchNCE}. In contrastive learning, a query patch is sampled from the sCT output and compared with the patch at the corresponding location (denoted as positive) or other patches at different locations (denoted as negatives) of the corresponding CBCT input image. The probability of the positive samples being selected over negatives can be formulated by the following cross-entropy loss:

$$l(v, v^+, v^-) = -\log \left[\frac{e^{v \cdot v^+/\tau}}{e^{v \cdot v^+/\tau} + \sum_{n=1}^{N} e^{v \cdot v_n^-/\tau}} \right] \tag{S3}$$

where v, v^+ and v^- denote the K-dimensional vectors of the query ($v \in \mathbb{R}^K$), the positive ($v^+ \in \mathbb{R}^K$), and N number of negatives ($v^- \in \mathbb{R}^{N \times K}$), respectively. The n-th negative is denoted as $v_n^- \in \mathbb{R}^{N \times K}$. For such an (N+1) classification problem, τ, which denotes the distances between the query and samples was set as 0.07. The goal here is to maximize the mutual information between v and v^+, but minimize between v and v^-.

The images from the positive and negative samples are passed through the encoder network of the generator (G_{enc}) to obtain embeddings. These embeddings are low-dimensional representations of the images that capture their content and style information. The layers of interest (L) and the number of spatial locations in each layer (S) are

selected. The feature maps are passed through a small two-layer multi layer perceptron (MLP) network $\mathrm{H_l}$, yielding a stack of features $\{z_l\}_L = \{H_l(G^l_{enc}(x))\}_L$. Likewise, the output image is encoded with the same network into $\hat{z}^s_l = \{H_l(G^l_{enc}(G(x)))\}_L$. The other patches within the input can be used as negatives and that formulates the following contrastive loss $L_{\mathrm{PatchNCEx}}$.

$$L_{\mathrm{PatchNCEx}}(\mathrm{G,H,CBCT}) = \mathbb{E}_{\mathrm{x \sim CBCT}} \sum_{l=1}^{L} \sum_{s=1}^{S_l} l(\hat{z}^s_l, z^s_l, z^{S/s}_l) \tag{S4}$$

where $\hat{z}^s_l$, z^s_l and $z^{S/s}_l$ represents the feature of the output image, the corresponding feature ($z^s_l \in \mathbb{R}^{C_l}$) and the negative feature ($z^{S/s}_l \in \mathbb{R}^{(S_L-1)\times C_l}$). C_l denotes the number of channels at each layer. Since the generator learns to pay attention to the similarities between the two domains, the embeddings share the common features. To avoid incorrect anatomical changes, CT is used to generate identical CT using the same generator. The positive and negative samples of these CT images are passed through the same $\mathrm{G_{enc}}$ and MLP, which allows us to formulate the following contrastive loss for the CT domain $L_{\mathrm{PatchNCEy}}$:

$$L_{\mathrm{PatchNCEy}}(\mathrm{G,H,CT}) = \mathbb{E}_{\mathrm{y \sim CT}} \sum_{l=1}^{L} \sum_{s=1}^{S_l} l(\hat{z}^s_l, z^s_l, z^{S/s}_l) \tag{S5}$$

The total loss function is therefore

$$\begin{aligned} L_{\mathrm{CUT}}(\mathrm{G, D_{CT}, CBCT, CT}) = & L_{\mathrm{adv}}(\mathrm{G, D_{CT}, CBCT, CT}) + \\ & \lambda_{\mathrm{CBCT}} L_{\mathrm{PatchNCEx}}(\mathrm{G, H, CBCT}) + \\ & \lambda_{\mathrm{CT}} L_{\mathrm{PatchNCEy}}(\mathrm{G, H, CT})). \end{aligned} \tag{S6}$$

where λ_{cbct} and λ_{ct} were both set to 10 for the optimal image quality as suggested in [7]. The main objective for CUT here is to generate realistic CT images, while patches in the input and output images share corresponding information.

Hyper-parameters and network settings

In the following, we describe the hyper-parameters and the network settings for each model.

- cycleGAN: a majority of the hyper-parameters which gave the best results in our previous study [1] were used, i.e. for the generators, two convolutional layers with stride 2 in the encoder and two deconvolutional layers with stride 2 in the decoder, nine residual blocks [8] between encoding and decoding operations. For the discriminators, we used 70 × 70 PatchGAN [9] with a downsampling scheme from 256 × 256 to 32 × 32 by applying four series of 2D convolutional layers, followed by instance normalization (not for the first and last layer) and LeakyReLU with a slope of 0.2 as nonlinearity (not for the last layer). The receptive field of the network was 70 × 70 and each pixel in the output was evaluated as a scalar in the range [0, 1]. Both generators and discrimninators were optimized with the Adam algorithm. The learning rate was set to 0.0002 during the first 100 epochs, and gradually reduced to zero over the next 100 epochs. The batch size was set to one.

- CUT: the hyper-parameters which were provided in the original CUT implementation [10] were mainly employed. Similar to the network architecture and hyperparameters of cycleGAN, we used the identical nine residual blocks and the PatchGAN discriminator, batch size of one, Adam optimizer with initial learning rate 0.0002 for first 200 epochs and then reduced linearly to zero over the next 200 epochs. Same as in cycleGAN, the hyper-parameters β_1 and β_2 that were used to calculate the momentum term of Adam were set as 0.5 and 0.999 respectively. For the L_{PatchNCE}, 5 layers of features were extracted (L=5), corresponding to the receptive fields of sizes 1 × 1, 9 × 9, 15 × 15, 35 × 35, and 99 × 99. For every layer's features, 256 random locations (S = 256) were sampled, and a 2-layer MLP was used to acquire final features.

An identical geometric augmentation pipeline was employed in both algorithms as described in [1]. Each CBCT and vCT input image was resampled to 256 × 256 pixels, followed by two dimensional (2D) horizontal flipping and affine transformations including rotation of [-5°, 5°] and scaling by [0.9, 1.1] with a bicubic interpolation over 4 × 4 neighboring pixels.

Implementation details

All models were trained and evaluated using the PyTorch based framework MONAI 1.1.0. [11] on an NVIDIA RTX A6000 GPU with 48 GB of memory.

Supplementary Figure 1

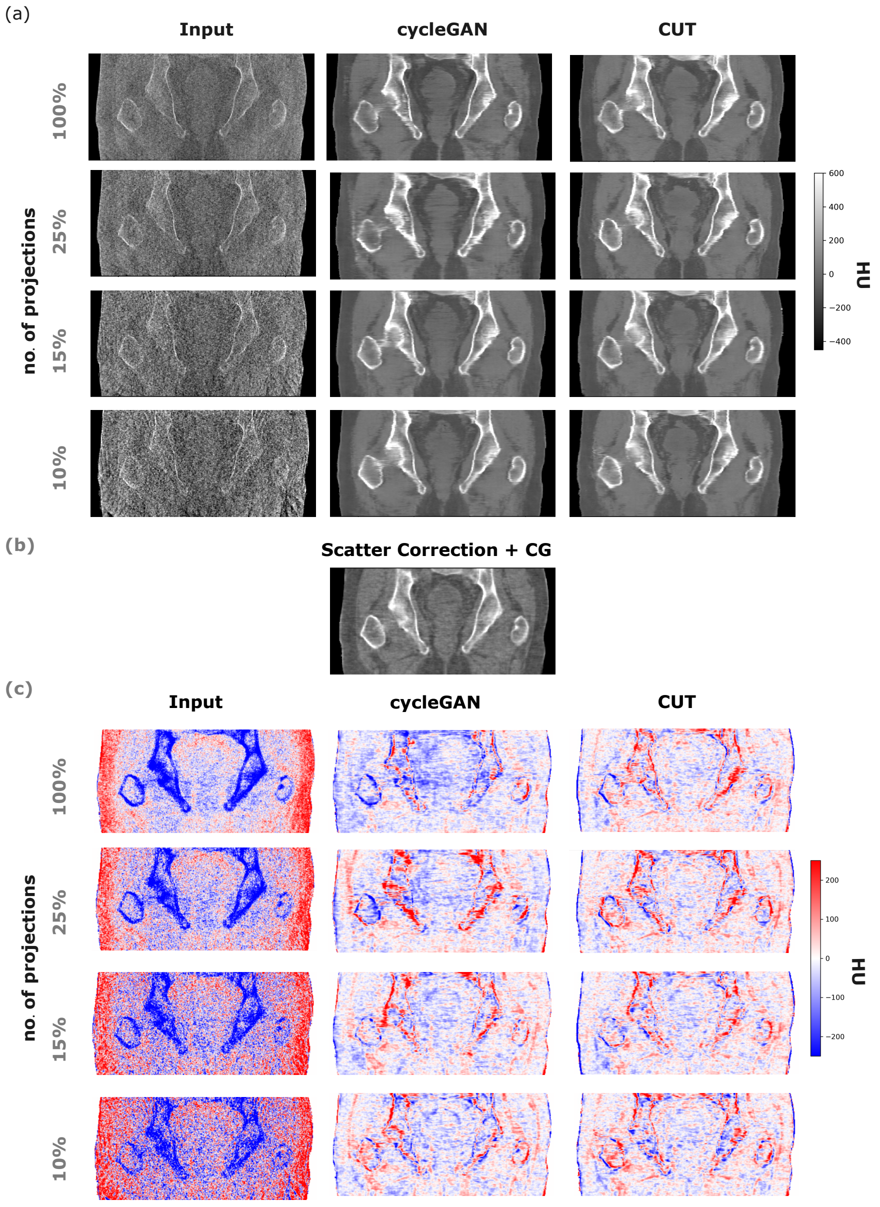

Figure S1: Coronal view of (a) the CBCT inputs and sCTs generated by cycleGAN and CUT with 100%, 25%, 15%, 10% projections; (b) the scatter corrected $\mathrm{CBCT_{cor}}$ reconstructed with conjugate gradient; (c) HU difference between corresponding sCTs and $\mathrm{CBCT_{cor}}$ of a test patient.

Supplementary Table 1

Table S1: Best epoch selection and the corresponding training time

	cycleGAN				CUT			
**No. of Projections**	100%	25%	15%	10%	100%	25%	15%	10%
Best epoch	40	60	150	160	250	360	340	350
Training time (hours)	7	10	25	27	42	60	57	58

Supplementary Table 2

Table S2: Average HU mean absolute error and mean error of test patient set for the comparison of sCTs and CBCT inputs with $CBCT_{cor}$, respectively. The number in square brackets represent [min, max] values among all patients in the corresponding groups. CBCT inputs were reconstructed using FDK without correction.

Mean Absolute Error (HU)

**Dataset**	**100%**	**25%**	**15%**	**10%**
**cycleGAN**	54 [48, 66]	56 [49, 65]	58 [52, 68]	59 [54, 68]
**CUT**	49 [44, 60]	51 [46, 61]	52 [46, 63]	54 [48, 65]
**CBCT inputs**	102 [97, 108]	120 [115, 126]	144 [137, 152]	164 [158, 173]

Mean Error (HU)

**Dataset**	**100%**	**25%**	**15%**	**10%**
**cycleGAN**	-18 [-35, -6]	-2 [-23, 13]	-21 [-39, -8]	-5 [-8, 16]
**CUT**	-5 [-12, 6]	-2 [-10, 5]	-9 [-17, -2]	-5 [-15, 4]
**CBCT inputs**	15 [-10, 33]	15 [-9, 32]	16 [10, 34]	18 [-8, 36]

Structural Similarity Index Measure

**Dataset**	**100%**	**25%**	**15%**	**10%**
**cycleGAN**	0.96 [0.94, 0.97]	0.95 [0.92, 0.97]	0.94 [0.92, 0.96]	0.94 [0.91, 0.95]
**CUT**	0.96 [0.93, 0.97]	0.95 [0.92, 0.97]	0.95 [0.92, 0.96]	0.94 [0.91, 0.96]
**CBCT inputs**	0.91 [0.87, 0.93]	0.87 [0.81, 0.89]	0.83 [0.77, 0.86]	0.81 [0.75, 0.84]

Peak Signal-to-Noise Ratio (dB)

**Dataset**	**100%**	**25%**	**15%**	**10%**
**cycleGAN**	36 [34, 39]	35 [33, 38]	34 [32, 36]	33 [31, 36]
**CUT**	36 [34, 39]	36 [33, 38]	35 [33, 37]	34 [32, 37]
**CBCT inputs**	33 [30, 35]	31 [29, 34]	29 [27, 32]	28 [26, 30]

Supplementary Table 3

Table S3: Mean absolute transformation parameter differences comparing sCTs-to-pCT from each dose levels with $CBCT_{FS}$-to-pCT for all test patients.

	**cycleGAN**				**CUT**			
**No. of Projections**	100%	25%	15%	10%	100%	25%	15%	10%
Right-left (mm)	0.03	0.02	0.03	0.03	0.03	0.03	0.03	0.02
Inferior-superior (mm)	0.04	0.05	0.04	0.04	0.04	0.06	0.05	0.05
Posterior-anterior (mm)	0.02	0.03	0.02	0.03	0.03	0.03	0.02	0.02
Pitch (°)	0.10	0.18	0.15	0.17	0.17	0.17	0.21	0.24
Roll (°)	0.14	0.15	0.17	0.19	0.16	0.18	0.15	0.18
Yaw (°)	0.23	0.25	0.20	0.20	0.26	0.26	0.24	0.19

References

[1] Chan Y, Li M, Parodi K, Belka C, Landry G, Kurz C. Feasibility of CycleGAN enhanced low dose CBCT imaging for prostate radiotherapy dose calculation. Phys Med Biol 2023;68:105014. https://doi.org/10.1088/1361-6560/acccce.

[2] Hofmaier J, Haehnle J, Kurz C, Landry G, Maihoefer C, Schüttrumpf L et al. Multi-criterial patient positioning based on dose recalculation on scatter-corrected CBCT images. Radiother Oncol 2017;125:464–469. https://doi.org/10.1016/j.radonc.2017.09.020.

[3] Park YK, Sharp GC, Phillips J, Winey BA. Proton dose calculation on scatter-corrected CBCT image: feasibility study for adaptive proton therapy. Med Phys 2015;42:4449–4459. https://doi.org/10.1118/1.4923179.

[4] Kurz C, Kamp F, Park YK, Zöllner C, Rit S, Hansen D et al. Investigating deformable image registration and scatter correction for CBCT-based dose calculation in adaptive impt. Med Phys 2016;43:5635–5646. https://doi.org/10.1118/1.4962933.

[5] Rit S, Oliva MV, Brousmiche S, Labarbe R, Sarrut D, Sharp GC. The Reconstruction Toolkit (RTK), an open-source cone-beam CT reconstruction toolkit based on the Insight Toolkit (ITK). J Phys Conf Ser 2014;489:012079. https://doi.org/10.1088/1742-6596/489/1/012079.

[6] de Bel T, Bokhorst JM, van der Laak J, Litjens G. Residual cycleGAN for robust domain transformation of histopathological tissue slides. Medical Image Analysis 2021;70:102004.

[7] Dong G, Zhang C, Deng L, Zhu Y, Dai J, Song L et al. A deep unsupervised learning framework for the 4D CBCT artifact correction. Phys Med Biol 2022;67:055012. https://doi.org/10.1088/1361-6560/ac55a5.

[8] Johnson J, Alahi A, Fei-Fei L. Perceptual losses for real-time style transfer and super-resolution. European conference on computer vision 2016;:694–711.

[9] Isola P, Zhu JY, Zhou T, Efros AA. Image-to-image translation with conditional adversarial networks. in: Proceedings of the IEEE conference on computer vision and pattern recognition, 2017, pp. 1125–1134.

[10] Park T, Efros AA, Zhang R, Zhu JY. Contrastive learning for unpaired image-to-image translation. Comput. Vis. ECCV 2020;12354:319–345. https://doi.org/10.48550/arXiv.2007.15651.

[11] Cardoso MJ, Li W, Brown R, Ma N, Kerfoot E, Wang Y et al. MONAI: An open-source framework for deep learning in healthcare 2022;:. https://doi.org/10.48550/arXiv.2211.02701.
